# Response: Use of the WISCI-II score in assessing outcome of intensive robot-assisted gait training in spinal cord injury

**DOI:** 10.1038/s41393-024-01044-y

**Published:** 2024-10-28

**Authors:** D. J. Edwards, A. Jayaraman

**Affiliations:** 1 Jefferson Moss Rehabilitation Research Institute & Department of Rehabilitation Medicine, Thomas Jefferson University, Philadelphia, PA USA; 2 School of Medical and Health Sciences & Exercise Medicine Research Institute, Edith Cowan University, Perth, WA Australia; 3 Max Nader Center for Rehabilitation Technologies & Outcomes Research, Shirley Ryan AbilityLab, Chicago, IL USA; 4 Department of Physical Medicine and Rehabilitation, Northwestern University’s Feinberg School of Medicine, Chicago, IL USA

The Walking Index in Spinal Cord Injury II (WISCI-II) assesses physical assistance and use of devices necessary during a 10 m walking test and is a useful tool for understanding the impact of intensive gait training on gait function. The primary outcome of clinical gait trials often hinges on a change in gait speed or endurance, yet other clinically relevant outcomes such as level of physical assistance and devices required, should also be carefully examined. In the WISE trial [1] the testing conditions for the gait speed assessment (primary outcome measure) were consistent pre and post intervention, such that any change in speed might reasonably be attributed to the change in gait ability, rather than a consequence of the change in external support between the two assessment time points. The WISCI-II, as well as a range of relevant secondary outcome measures were assessed. The WISE trial analyses showed no statistical difference within or between groups on WISCI-II score changes across time points. Since the WISCI-II score (a) did not change in any group, and (b) encompasses both physical assistance as well as change in use of devices, we interpreted that there was no change in the context of our study design and sample size. We reported a few cases of change in devices for interest to the reader to highlight that there was no clear pattern of response across groups. The Correspondence by Dr Selim [2] is astute and the authors agree that future studies enroling SCI participants who can stand and step should consider the WISCI-II as a key outcome measure given its functional relevance. The statistical design will be important, and where appropriate, investigators may report on changes at the individual level for both physical assistance and the use of devices. Ditunno et al [3] discuss the appropriate use of this scale. Ultimately, gait function improvements from intervention can involve an interaction of speed, physical assistance and device support, which may be differentially implicated depending on an individual’s level of function, and study findings should be interpreted in this light.
